# MR-proANP and incident cardiovascular disease in patients with type 2 diabetes with and without heart failure with preserved ejection fraction

**DOI:** 10.1186/s12933-020-01155-9

**Published:** 2020-10-16

**Authors:** Jesper Jensen, Morten Schou, Caroline Kistorp, Jens Faber, Tine W. Hansen, Magnus T. Jensen, Henrik U. Andersen, Peter Rossing, Tina Vilsbøll, Peter G. Jørgensen

**Affiliations:** 1 Department of Cardiology, Herlev and Gentofte Hospital, Borgmester Ib Juuls Vej 1, 2730 Herlev, Denmark; 2 Department of Endocrinology, Rigshospitalet, Blegdamsvej 9, 2100 Copenhagen, Denmark; 3 Department of Endocrinology, Herlev and Gentofte Hospital, Borgmester Ib Juuls Vej 1, 2730 Herlev, Denmark; 4 Steno Diabetes Center Copenhagen, Niels Steensens Vej 2, 2820 Gentofte, Denmark; 5 Department of Cardiology, Copenhagen University Hospital Amager-Hvidovre Hospital, Kettegård Alle 30, 2650 Hvidovre, Denmark; 6 Faculty of Health and Medical Sciences, Copenhagen University, Blegdamsvej 3B, 2200 Copenhagen, Denmark

**Keywords:** Cardiovascular disease, Diabetes complications, Macrovascular disease, Type 2 diabetes, MR-proANP, Heart failure

## Abstract

**Background:**

Mid-regional pro-atrial natriuretic peptide (MR-proANP) is a useful biomarker in outpatients with type 2 diabetes (T2D) to diagnose heart failure (HF). Elevated B-type natriuretic peptides are included in the definition of HF with preserved ejection fraction (HFpEF) but little is known about the prognostic value of including A-type natriuretic peptides (MR-proANP) in the evaluation of patients with T2D.

**Methods:**

We prospectively evaluated the risk of incident cardiovascular (CV) events in outpatients with T2D (n = 806, mean ± standard deviation age 64 ± 10 years, 65% male, median [interquartile range] duration of diabetes 12 [6–17] years, 17.5% with symptomatic HFpEF) according to MR-proANP levels and stratified according to HF-status including further stratification according to a prespecified cut-off level of MR-proANP.

**Results:**

A total of 126 CV events occurred (median follow-up 4.8 [4.1–5.3] years). An elevated MR-proANP, with a cut-off of 60 pmol/l or as a continuous variable, was associated with incident CV events (p < 0.001). Compared to patients without HF, patients with HFpEF and high MR-proANP (≥ 60 pmol/l; median 124 [89–202] pmol/l) and patients with HF and reduced ejection fraction (HFrEF) had a higher risk of CV events (multivariable model; hazard ratio (HR) 2.56 [95% CI 1.64–4.00] and 3.32 [1.64–6.74], respectively). Conversely, patients with HFpEF and low MR-proANP (< 60 pmol/l; median 46 [32–56] pmol/l) did not have an increased risk (HR 2.18 [0.78–6.14]).

**Conclusions:**

Patients with T2D and HFpEF with high MR-proANP levels had an increased risk for CV events compared to patients with HFpEF without elevated MR-proANP and compared to patients without HF, supporting the use of MR-proANP in the definition of HFpEF from a prognostic point-of-view.

## Background

The development of heart failure (HF) in patients with type 2 diabetes (T2D) worsens prognosis dramatically [1]. Especially HF with preserved ejection fraction (HFpEF) is frequent in these patients [2]. However, the diagnosis of HFpEF is challenging, and more knowledge on HFpEF in patients with T2D is needed [3]. In the most recent HF guidelines from the European Society of Cardiology (ESC), the diagnosis of HFpEF includes signs and/or symptoms of HF, echocardiographic criteria, and increased natriuretic peptides [4]. Natriuretic peptides are sensitive markers of hemodynamic status and elevated levels might represent an unstable state of HFpEF, that is, a maladapted cardiac remodeling leading to subsequent cardiovascular (CV) events [5]. However, only B-type natriuretic peptides (B-type natriuretic peptide (BNP) and N-terminal proBNP (NT-proBNP)) are included in the HFpEF diagnosis and data on A-type natriuretic peptides (mid-regional pro-atrial natriuretic peptide (MR-proANP)) are lacking [6]. In a recent position paper from the ESC HF association, measurement of natriuretic peptides in high-risk populations such as patients with T2D was recommended, but again, evidence is primarily existing on B-type natriuretic peptides, and more data on A-type natriuretic peptides are needed [7]. A-type natriuretic peptides could carry similar relevant prognostic information when included in the HFpEF definition, as has previously been shown for B-type natriuretic peptides [4]. In the acute setting, MR-proANP has shown to improve the diagnostic performance of B-type natriuretic peptides for HF in obese patients [8]. Also, MR-proANP has been shown to be associated with clinical outcomes in HFpEF, not HF with reduced ejection fraction (HFrEF), [9] and in an at-risk community population, NT-proBNP and MR-proANP have shown to predict incident HF [10]. Moreover, MR-proANP levels have shown similar diagnostic performance as NT-proBNP in primary care patients suspected of non-acute HF [11]. And in a high-risk population such as outpatients with T2D, our group recently reported, that MR-proANP with a cut-off of less than 60 pmol/l was efficient to rule out HFrEF and at this cut-off, the highest ability to similarly rule out HFpEF was obtained. However, the accuracy to rule out HFpEF was still relatively low. Despite having a relatively low diagnostic value in HFpEF, the prognostic value of MR-proANP in HFpEF could potentially guide the diabetologist in identifying patients with a high risk of future clinical events, in which further cardiac work up should be performed and relevant treatment started [2]. In the present study, we sought to evaluate the prognostic value of MR-proANP and examine the significance of HFpEF in the presence and absence of elevated MR-proANP levels using the pre-specified cut-off level.

## Methods

The study was conducted in accordance with the Helsinki Declaration and approved by the Danish National Committee on Biomedical Research Ethics (amendment to protocol no. H‐3‐2009‐139) [12].

### Study population

The study population consisted of patients with T2D from the Thousand&2 Study, which has previously been described in detail [13]. In brief, patients followed at two specialized diabetes clinics in Denmark, the Center for Diabetes research (CfD) at Herlev‐Gentofte University Hospital and the Steno Diabetes Center Copenhagen (SDCC), were eligible to participate [13]. A total of 1030 patients were included in the Thousand&2 Study (participation rate 47.8%). Of these, two hundred and twenty-four patients with either prior heart valve replacement, atrial fibrillation and/or missing laboratory values/echocardiographic measurements were excluded rendering 806 patients available for the present study [2].

### Baseline study visit, biochemistry and echocardiograms

The baseline study visit, biochemistry and echocardiograms have previously been described in detail [2, 14]. In brief, all study related data were collected at or in close conjunction with the baseline study visit including blood samples for the study biobank and the echocardiographic assessments. After immediate centrifugation, blood samples were stored at − 80 °C and MR-proANP was analyzed in a single batch in July 2017 using the KRYPTOR assay (BRAHMS GmbH/Thermo Fisher Scientific, Hennigsdorf, Germany) [2]. Echocardiograms were primarily (> 95%) performed and analyzed by a single investigator (P.G.J.) in accordance with the existing European and American guidelines as previously described [14, 15]. Albuminuria was defined as urine albumin/creatinine ratio above 30 mg/g or urine albumin above 30 mg/day on at least 2 consecutive measurements [13].

### Heart failure definitions

Based on the current European and American guidelines we defined HF as follows: HFpEF was defined as self-reported dyspnea corresponding to the New York Heart Association (NYHA) functional class II–IV and presence of at least one of the following echocardiographic findings (a–d): (a) left ventricular ejection fraction (LVEF) > 40% and ≤ 50%, (b) ratio of early diastolic mitral inflow velocity (E) to early diastolic septal annular velocity (e′) (E/e′_septal_) ≥ 15, (c) increased left ventricular (LV) mass index (> 95 g/cm^2^ for women and > 115 g/cm^2^ for men), and (d) left atrial volume index > 34 mL/m^2^. HFrEF was defined as a LVEF ≤ 40%, regardless of reported dyspnea. The definitions were specified before the MR-proANP analyses were performed.

### Follow-up

Information on incident CV events were retrieved through national registries. A CV event was defined as the composite of admission with CV disease (including HF, coronary revascularization, myocardial infarction, cardiac arrest, cerebrovascular disease and peripheral artery disease) and CV death.

### Statistics

Baseline characteristics for all four groups were compared using a one-way analysis of variance for continuous variables and, in case of non-normal distribution, Mann–Whitney U tests or Kruskall-Wallis tests were used. Categorical variables were compared using the Chi-square test. MR-proANP levels were non-normally distributed and, therefore, log2-transformed before continuous analyses. Cumulative incidence curves with non-CV death as competing risk for incident CV events according to HF status were performed, and for HFpEF also according to the prespecified dichotomized MR-proANP level of 60 pmol/l. Moreover, a cumulative incidence curve for incident CV events according to the dichotomized MR-proANP level was performed with non-CV death as competing risk. Poisson regression with restricted cubic splines was used to examine the association of incidence rates and MR-proANP levels. The Akaike information criterion was used to determine the optimal number of knots. A multivariable Cox proportional hazards model was constructed including the prespecified variables age, sex, duration of diabetes, known CV disease, uncontrolled systolic blood pressure (SBP) above 170 mmHg, body mass index (BMI) and albuminuria. The performance of the multivariable model was assessed by C-statistics and continuous net reclassification index (NRI) with and without HF or MR-proANP in the model. Associations between MR-proANP levels and the echocardiographic measures were analyzed. A p-value less than 0.05 (two-sided) was considered significant. Statistics were calculated using R for Mac, version 3.4.2 (R Project for Statistical Computing, Vienna University of Economics and Business administration, Austria).

## Results

### Baseline characteristics

Baseline characteristics for the 806 included patients are presented in Table 1. Compared to patients with HFpEF and a low MR-proANP, patients with HFpEF and a high MR-proANP were characterized by being older, with a higher frequency of males, a higher prevalence of known ischemic heart disease, a higher creatinine level and were to a higher extend treated with betablockers, diuretics and anti-platelet therapy. Generally, patients with HFpEF and a high MR-proANP were similar to patients with HFrEF. During a median [interquartile range (IQR)] follow-up of 4.8 [4.1–5.3] years, a total of 126 incident CV events occurred in the total cohort.

**Table 1:** Baseline characteristics according to heart failure status

	No HF	HFpEF low MR-proANP	HFpEF high MR-proANP	HFrEF	p-value
n	646	27	114	19	
Age, year (median [IQR])	64.4 [56.9–69.8]	60.2 [53.0–65.7]	69.8 [64.7–74.9]	72.2 [64.9–76.6]	< 0.001
Male, n (%)	433 (67.0)	10 (37.0)	67 (58.8)	15 (78.9)	0.003
Body mass index, kg/m^2^ (median [IQR])	28.7 [26.0–32.8]	31.8 [28.9–38.2]	31.6 [28.0–34.2]	28.4 [26.0–31.3]	< 0.001
T2DM duration, year (median [IQR])	11.0 [5.0–16.0]	15.0 [8.0–23.5]	15.0 [7.3–20.8]	12.0 [10.0–23.0]	< 0.001
Known ischemic heart disease, n (%)	97 (15.0)	2 (7.4)	42 (36.8)	9 (47.4)	< 0.001
Smoking, n (%)					0.903
Never	276 (42.7)	13 (48.1)	47 (41.2)	8 (42.1)	
Active	88 (13.6)	5 (18.5)	19 (16.7)	2 (10.5)	
Former	282 (43.7)	9 (33.3)	48 (42.1)	9 (47.4)	
Systolic blood pressure, mmHg (mean (sd))	134.6 (16.2)	134.8 (15.7)	139.1 (20.0)	141.3 (18.6)	0.029
Diastolic blood pressure, mmHg (mean (sd))	80.0 (10.8)	79.8 (10.1)	77.8 (10.9)	81.6 (9.3)	0.226
Heart rate, bpm (mean (sd))	72.4 (11.2)	76.6 (9.7)	68.2 (10.8)	72.7 (11.6)	< 0.001
Angina, n (%)	44 (7.0)	6 (22.2)	23 (20.7)	4 (22.2)	< 0.001
Dyspnea, n (%)	162 (25.1)	27 (100)	114 (100)	8 (42.1)	< 0.001
New York Heart Association class, n (%)					< 0.001
I	484 (74.9)	0 (0.0)	0 (0.0)	11 (57.9)	
II	117 (18.1)	13 (48.1)	66 (57.9)	5 (26.3)	
III	38 (5.9)	14 (51.9)	43 (37.7)	3 (15.8)	
IV	7 (1.1)	0 (0.0)	5 (4.4)	0 (0.0)	
MR-proANP, pmol/l (median [IQR])	65 [42–99]	46 [32–56]	124 [89–202]	125 [94–209]	< 0.001
LDL cholesterol, mmol/l (median [IQR])	2.0 [1.5–2.6]	2.1 [1.8–2.6]	1.9 [1.6–2.3]	2.1 [1.8–2.9]	0.468
Creatinine, μmol/l (median [IQR])	77.0 [65.0–93.0]	69.5 [60.3–76.8]	97.0 [77.0–123.0]	78.0 [66.5–96.0]	< 0.001
Albuminuria, n (%)	153 (23.7)	5 (18.5)	37 (32.5)	6 (31.6)	0.077
HbA1c, mmol/mol (median [IQR])	55.0 [48.0–65.0]	55.0 [48.5–71.0]	54.0 [48.0–67.0]	53.0 [44.5–69.0]	0.777
Hemoglobin, mmol/L (mean (SD))	8.7 (0.9)	8.4 (0.8)	8.1 (1.0)	8.8 (0.9)	< 0.001
Treatment with					
Metformin, n (%)	488 (75.5)	17 (63.0)	64 (56.1)	11 (57.9)	< 0.001
Dipeptidyl peptidase-4 inhibitor, n (%)	69 (10.7)	4 (14.8)	9 (7.9)	1 (5.3)	0.586
Sulfonylurea, n (%)	106 (16.4)	2 (7.4)	16 (14.0)	5 (26.3)	0.335
Glucagon-like peptide-1 receptor agonist, n (%)	155 (24.0)	7 (25.9)	18 (15.8)	3 (15.8)	0.222
Insulin, n (%)	275 (42.6)	17 (63.0)	68 (59.6)	13 (68.4)	< 0.001
Beta-blocker, n (%)	129 (20.0)	5 (18.5)	54 (47.4)	10 (52.6)	< 0.001
ACE inhibitor, n (%)	256 (39.6)	6 (22.2)	37 (32.5)	10 (52.6)	0.081
Angiotensin-II receptor blocker, n (%)	241 (37.3)	10 (37.0)	56 (49.1)	7 (36.8)	0.123
Calcium channel blocker, n (%)	200 (31.0)	10 (37.0)	44 (38.6)	5 (26.3)	0.364
Diuretics^a^, n (%)	297 (46.0)	7 (25.9)	77 (67.5)	11 (57.9)	< 0.001
Statins, n (%)	508 (78.6)	22 (81.5)	88 (77.2)	12 (63.2)	0.419
Anti-platelet therapy, n (%)	423 (65.5)	16 (59.3)	90 (78.9)	15 (78.9)	0.019

For patients with HFpEF, stratification according to low (< 60 pmol/l) or high MR-proANP level is presented. p-value for comparison of all four groups

ACE, angiotensin converting enzyme; bpm, beats per minute; HbA1c, glycated hemoglobin; HFpEF, heart failure with preserved ejection fraction; HFrEF, heart failure with reduced ejection fraction; IQR, interquartile range; LDL, low density lipoprotein; mm Hg, millimeters of mercury; MR-proANP, mid-regional pro-atrial natriuretic peptide; sd, standard deviation; T2D, type 2 diabetes mellitus

^a^Include loop diuretics, thiazides, or mineralocorticoid receptor antagonists

### Incidences of CV events according to HF status

**Fig. 1:**
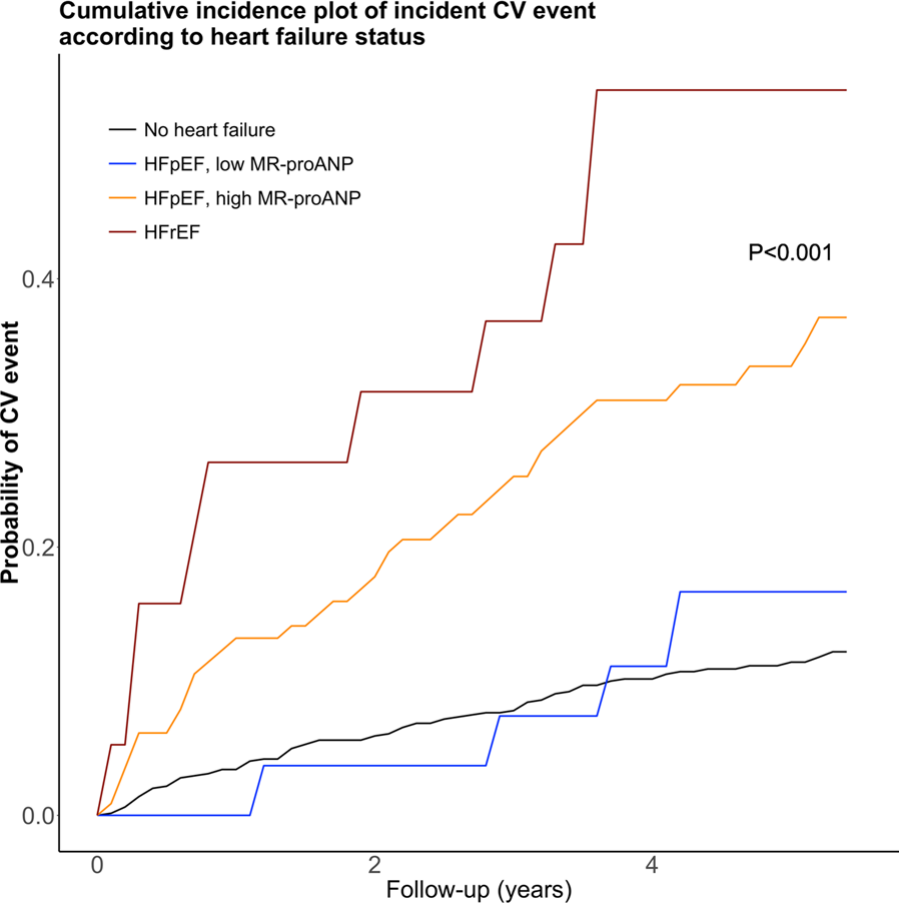
Cumulative incidence plot of incident cardiovascular events according to HF status. For patients with HFpEF, stratification according to low (< 60 pmol/l) or high MR-proANP level is presented. CV, cardiovascular; HFpEF, heart failure with preserved ejection fraction; HFrEF, heart failure with reduced ejection fraction; MR-proANP, mid-regional pro-atrial natriuretic peptide

**Fig. 2:**
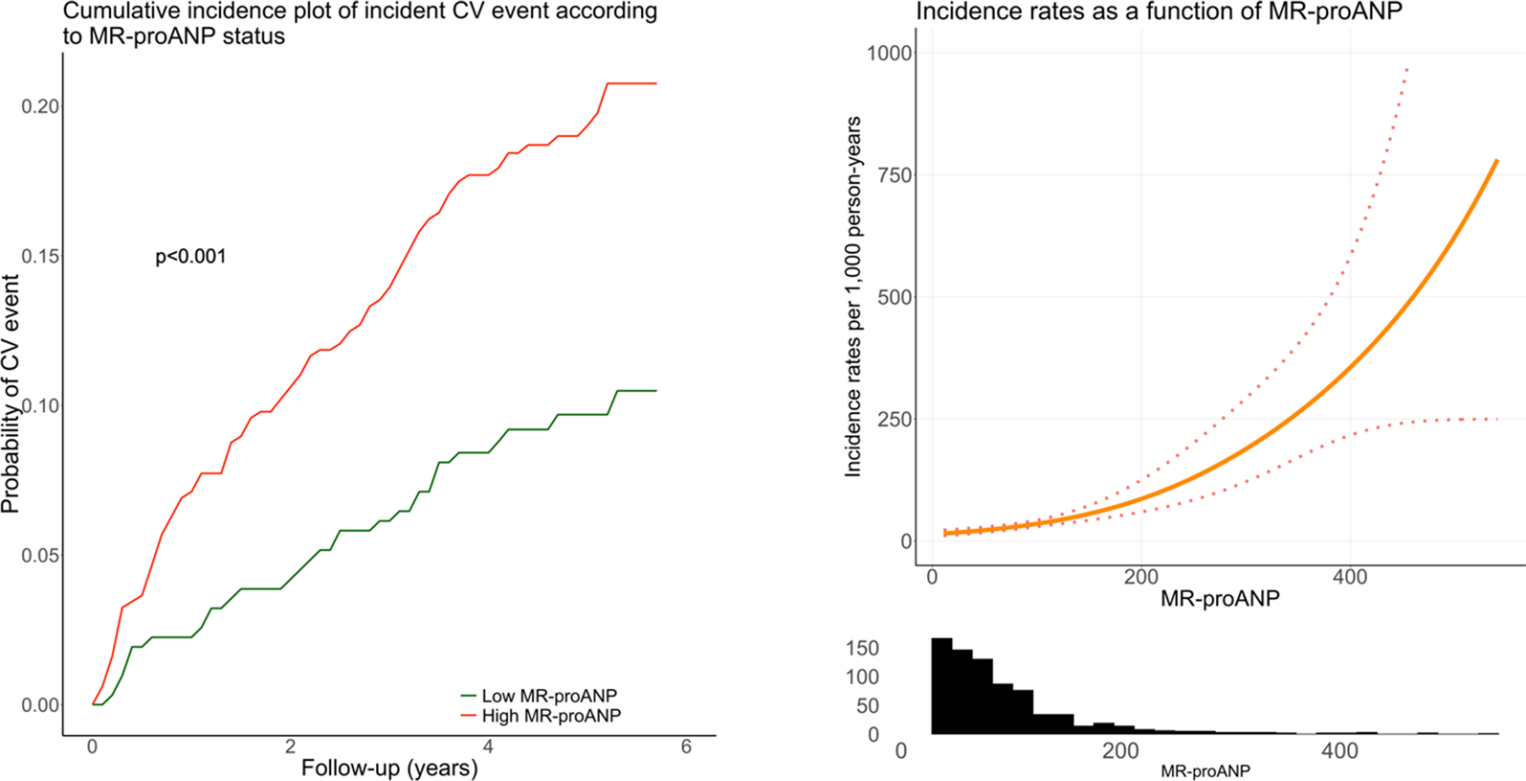
Cumulative incidence plot of incident CV event and incidence rates according to MR-proANP level. CV, cardiovascular; MR-proANP, mid-regional pro-atrial natriuretic peptide

**Fig. 3:**
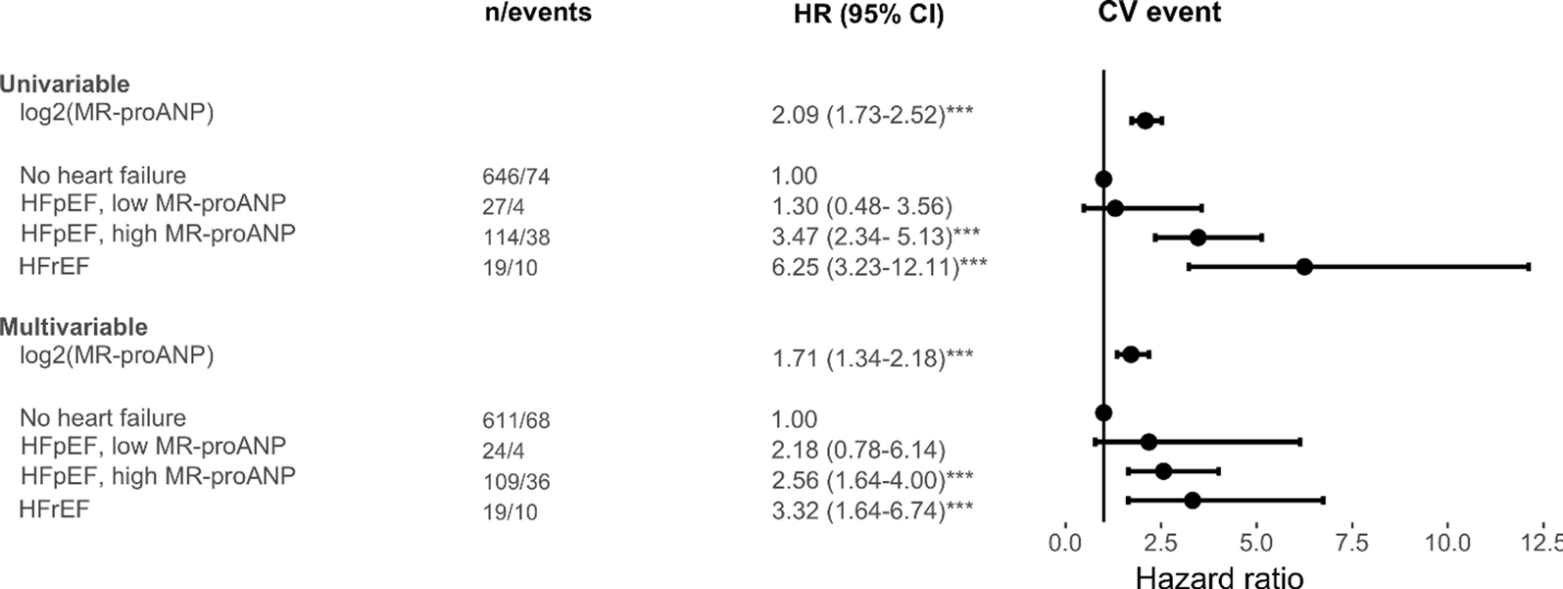
Forest plot according to HF status, in the univariable analysis and in the multivariable model. ***p < 0.001. CV, cardiovascular; HFpEF, heart failure with preserved ejection fraction; HFrEF, heart failure with reduced ejection fraction; MR-proANP, mid-regional pro-atrial natriuretic peptide

Patients with HFrEF, followed by patients with HFpEF and a high MR-proANP level, had the highest incidence of CV events (Fig. 1). Notably, patients with HFpEF and a low MR-proANP had an incidence of CV events, which was not statistically different to the incidence in patients without HF (Fig. 1). MR-proANP levels were associated with incident CV events; both analyzed as a dichotomized and a continuous variable (Fig. 2). In the univariable analysis and in the multivariable model, a higher MR-proANP level was associated with a higher risk of an incident CV event, with a hazard ratio (HR) [95% confidence interval (CI)] of 2.09 [1.73–2.52] and 1.71 [1.34–2.18] per doubling of MR-proANP, p < 0.001, respectively (Fig. 3). Patients with HFpEF and a low MR-proANP did not have a significantly different risk for an incident CV event compared to patients without HF (univariable analysis; HR 1.30 [0.48–3.56], multivariable model; HR 2.18 [0.78–6.14], p > 0.05) (Fig. 3). In contrast, the risk was significantly increased in both patients with HFpEF and high MR-proANP (univariable analysis; HR 3.47 [2.34–5.13], multivariable model; HR 2.56 [1.64–4.00], p < 0.001) and in patients with HFrEF (univariable analysis; HR 6.25 [3.23–12.11], multivariable model; HR 3.32 [1.64–6.74], p < 0.001) (Fig. 3). A sensitivity analysis with adjustment for SBP as a continuous variable instead of adjustment for SBP > 170 mmHg and with further adjustment for estimated glomerular filtration rate (eGFR) in addition to the prespecified variables showed overall similar results. Similarly, a sensitivity with adjustment for HbA1c instead of duration of type 2 diabetes in the prespecified model, also showed overall similar results (data not shown). In a competing risks regression model with the prespecified variables, sub-hazard ratios showed similar results as the traditional hazard ratios (Additional file 1: Table S1).

### Model performance

Results of the prognostic performance of models with MR-proANP and HF status alone and added to the multivariable adjusted model are shown Table 2. An MR-proANP level with a cut-off of 60 pmol/l had only limited prognostic performance assessed by c-statistics, 0.57 (0.54–0.62). MR-proANP as a continuous variable had a c-statistic of 0.66 (0.61–0.71), which was lower than the multivariable adjusted model alone, 0.72 (0.68–0.78). Adding MR-proANP as a continuous variable to the multivariable model, resulted in a significant increase in NRI of 27.1% [7.6–46.6], p = 0.007, and adding HF status showed an NRI of 32.8% [13.5–52.0], p < 0.001.

**Table 2:** Model performance

	C-statistics	Net reclassification index	p-value
MR-proANP			
Continuous variable (univariable)	0.66 (0.61–0.71)		
Cut-off 60 pmol/l (univariable)	0.57 (0.54–0.62)		
Multivariable analyses			
Model 1	0.72 (0.68–0.78)		
Model 1 + MR-proANP (continuous)	0.74 (0.69–0.78)	27.1% (7.6–46.6)	0.007
Model 1 + MR-proANP (Cut-off 60 pmol/l)	0.73 (0.68–0.77)	12.1% (-7.0–0.31)	0.22
Heart failure status			
Heart failure status (univariable)	0.63 (0.58–0.67)		
Model 1 + heart failure status	0.75 (0.71–0.80)	32.8% (13.5–52.0)	< 0.001

Model 1 includes age, sex, duration of diabetes, known CV disease, SBP > 170 mmHg, BMI and albuminuria

The multivariable Cox proportional hazards model (Model 1) with NRI when adding MR-proANP as a continuous or dichomized variable or when adding heart failure status. For reference, the univariable Cox proportional hazards models with MR-proANP as a continuous or dichotomized variable, and with heart failure status are shown

NRI, net reclassification index; MR-proANP, Mid-regional pro-atrial natriuretic peptide; CV, cardiovascular; SBP, systolic blood pressure; BMI, body mass index

### Associations between MR-proANP levels and echocardiographic measures

As supplemental analyses, the associations between MR-proANP levels and the echocardiographic measures were analyzed in univariable and multivariable analyses (Additional file 1: Table S2). In the multivariable analyses, MR-proANP was significantly associated with left ventricular mass index, left atrial volume index, the E/A ratio, the E/e′_mean_ ratio, and with measures of left ventricular systolic function.

## Discussion

In this large cohort of patients with T2D in an outpatient, non-acute setting, we found that; (1) higher MR-proANP levels were highly associated with incident CV events; (2) MR-proANP levels effectively stratified patients with symptomatic HFpEF and predicted the outcome of incident CV events during almost five years of follow-up; (3) patients with symptomatic HFpEF and high MR-proANP had an increased risk of CV events compared to patients with HFpEF without elevated MR-proANP and compared to patients without HF; (4) adding MR-proANP as a continuous variable or adding HF status to a model with clinical risk markers significantly reclassified 27.1% and 32.8% respectively, but only resulted in limited improvements in C-statistics; (5) finally, MR-proANP was associated with key echocardiographic measures of both diastolic—including left atrial volume index—and systolic function.

In patients with HF, natriuretic peptides are established diagnostic and prognostic biomarkers, and also carry relevant prognostic information in other cardiovascular diseases [7, 16]. Natriuretic peptides act as a surrogate measure of the underlying left ventricular dysfunction in HF [17]. The ventricular dysfunction causes volume expansion and/or pressure overload, leading to increased stress in the atrial and ventricular walls and thereby an increased release of natriuretic peptides. Moreover, natriuretic peptides have also been associated with metabolic changes potentially modulating their property as prognostic markers beyond the indication of ventricular dysfunction [18]. In some series of patients with HF (22% with concomitant T2D), the more recently developed biomarker MR-proANP has outperformed the extensively studied biomarker NT-proBNP in the prediction of mortality, [19] and some data indicate that MR-proANP may be more sensitive and specific than NT-proBNP in diagnosing HFpEF [20]. In patients with T2D, the most common co-existing conditions that cause HF is coronary artery disease and hypertension, [21] and MR-proANP has previously been shown to be independently associated with CV mortality in both primary and secondary care patients with T2D [22, 23]. In 1100 outpatients with T2D prospectively followed in primary care in the Netherlands, MR-proANP as a continuous variable was independently associated with both CV and all-cause mortality during a follow-up of 10 years with adjusted HRs of 2.42 [1.74–3.38] and 2.23 [1.78–2.79], respectively, in a multivariable model adjusted for age, sex, smoking, BMI, SBP, duration of diabetes, serum creatinine level, cholesterol-to-high-density lipoprotein ratio, macrovascular complications, albuminuria, and the use of lipid-lowering and anti-hypertensive medications [23]. Similarly, in a prospective cohort of more than 700 outpatients with T2D in secondary care in Austria, a one standard deviation increase in MR-proANP was associated with a 1.85-fold [1.49–2.30] increase in the risk of the composite outcome of unplanned hospitalization for CV disease or death during a median follow-up of 15 months, in a multivariable model adjusted for NYHA functional class, age, serum creatinine level, low-density lipoprotein level, level of serum triglycerides, hemoglobin A1c, SBP and BMI [22]. In addition to key differences compared with the present study in characteristics of the cohorts, follow-up times and definitions of outcome, no stratification according to HF status based on echocardiography was performed in these previous studies. Stratification according to HF status enables the opportunity to link the MR-proANP levels to the underlying changes in cardiac structure seen in HF patients. In outpatients with T2D, HFrEF is infrequent and can be ruled out with a low MR-proANP level, [2] which is important as therapies have been shown to reduce both morbidity and mortality in patients with HFrEF irrespective of concomitant T2D [4]. Conversely, HFpEF is frequent in outpatients with T2D, [2] and it has been hypothesized that T2D-related processes can cause HFpEF by direct effects on cardiac structure, [24, 25] and these changes in cardiac structure have been shown to be associated with CV events and all-cause mortality [5]. In a recent study, in which 37% had concomitant T2D, MR-proANP was associated with clinical outcomes in HFpEF, not in HFrEF, supporting the use of MR-proANP as a prognostic marker in patients with HFpEF [9]. Also, the use of MR-proANP in patients with HFpEF is supported by the current data, with a strong association between MR-proANP levels and key echocardiographic measures of diastolic—including left atrial volume index—and systolic function which are impaired in these patients. Based on the prognostic data in this large cohort of outpatients with T2D, an MR-proANP level of 60 pmol/l can support the diabetologist in identifying patients with HFpEF and increased risk of CV events, thus acting as a guide to identify patients for further cardiac diagnostic work up; patients with dyspnea and an MR-proANP of 60 pmol/l or greater should be referred for an echocardiogram. At present, the identification of patients with HFpEF has no therapeutic consequence as no effective therapies exist, [26] but patients with HFpEF and increased risk of CV events are candidates for ongoing clinical trials.

The used definition of HFpEF in the present study was prespecified based on the current knowledge in the field. However, as no definitive consensus on the definition of HFpEF exists across guidelines and for inclusion in clinical trials, other definitions could have been valid, and this should be kept in mind when comparing the present results with other studies on HFpEF. Strengths of the present study include the comprehensive characterization of the patients at baseline including echocardiographic assessment. The MR-proANP levels were analyzed in a single batch, consequently reducing potential analytical variability, using a contemporary assay. As the aim was to specifically generate data on MR-proANP, no conclusions regarding potential differences to B-type natriuretic peptides can be made from the present data. The completeness of the registered CV events in the Danish national registries diminishes the possible misclassification of events. Conclusions regarding differences in outcomes between the groups are limited due to the low number of patients in the group with HFpEF and low MR-proANP and in the group with HFrEF, and larger studies are needed to investigate these possible differences. Additionally, the findings of this study should be tested in other cohorts to ascertain their robustness. The results of the present study cannot be extrapolated to non-diabetic patients or to patients with T2D and atrial fibrillation. Extrapolation to other races than Caucasians should be done with caution. Moreover, despite adjustments for clinically important confounders in the multivariable analyses, the risk of other factors confounding the results is still possible and should be kept in mind when interpreting the results of the present study. A strict cost–benefit analysis should be performed before implementation of routine MR-proANP measurements in the clinical setting.

## Conclusions

In conclusion, our study demonstrates that MR-proANP levels were highly associated with incident cardiovascular events in outpatients with type 2 diabetes and that patients with HFpEF and high MR-proANP levels had a higher risk for cardiovascular events compared to patients with HFpEF without elevated MR-proANP and compared to patients without heart failure. The present findings support the use of MR-proANP as a test in outpatients with type 2 diabetes and support the inclusion of MR-proANP in the definition of HFpEF from a prognostic point-of-view.

## Supplementary information

**Additional file 1: Table S1.** Competing risk analyses with Fine-Gray method with all-cause mortality as competing risk to CV event. **Table S2: **Associations between MR-proANP and echocardiographic measures.

## Data Availability

The datasets used and/or analyzed during the current study are available from the corresponding author on reasonable request.

## Abbreviations

ANP: A-type natriuretic peptide
BMI: Body mass index
BNP: B-type natriuretic peptide
CfD: Center for Diabetes research, Herlev‐Gentofte University Hospital
CI: Confidence interval
CV: Cardiovascular
E: Early diastolic mitral inflow velocity
e′_septal_: Early diastolic septal annular velocity
eGFR: Estimated glomerular filtration rate
ESC: European society of cardiology
HF: Heart failure
HFpEF: Heart failure with preserved ejection fraction
HFrEF: Heart failure with reduced ejection fraction
HR: Hazard ratio
IQR: Interquartile range
LV: Left ventricular
LVEF: Left ventricular ejection fraction
MR-proANP: Mid-regional pro-atrial natriuretic peptide
NRI: Net reclassification index
NT-ANP: N-terminal ANP
NT-proBNP: N-terminal pro-B type natriuretic peptide
NYHA: New York Heart Association
SBP: Systolic blood pressure
sd: Standard deviation
SDCC: Steno Diabetes Center Copenhagen
T2D: Type 2 diabetes
