# Supplementary material for: MR-proANP and incident cardiovascular disease in patients with type 2 diabetes with and without heart failure with preserved ejection fraction
Source: Cardiovasc Diabetol. 2020 Oct 16;19:180. doi: 10.1186/s12933-020-01155-9 (PMC7568388; doi:10.1186/s12933-020-01155-9)
Supplement: Supplementary file 1 — Additional file 1: Table S1. Competing risk analyses with Fine-Gray method with all-cause mortality as competing risk to CV event. Table S2: Associations between MR-proANP and echocardiographic measures. [file 12933_2020_1155_MOESM1_ESM.docx]

**Additional file 1: Table S1.** Competing risk analyses with Fine-Gray method with all-cause mortality as competing risk to CV event.

|  | |  | Sub-hazard ratio  (95% confidence interval) | P-value |
| --- | --- | --- | --- | --- |
| Univariable | |  |  |  |
|  | log2(MR-proANP) |  | 1.99 (1.63-2.42) | <0.001 |
|  |  |  |  |  |
|  | HFpEF, low MR-proANP |  | 1.53 (0.58-4.02) | 0.39 |
|  | HFpEF, high MR-proANP |  | 3.40 (2.27-5.09) | <0.001 |
|  | HFrEF |  | 6.23 (3.18-12.21) | <0.001 |
|  | |  |  |  |
| Multivariable | |  |  |  |
|  | log2(MR-proANP) |  | 1.66 (1.30-2.11) | <0.001 |
|  |  |  |  |  |
|  | HFpEF, low MR-proANP |  | 2.21 (0.81-6.05) | 0.12 |
|  | HFpEF, high MR-proANP |  | 2.47 (1.56-3.93) | <0.001 |
|  | HFrEF |  | 1.20 (1.55-7.11) | 0.002 |

Multivariable model adjusted for age, sex, BMI, diabetes duration, systolic blood pressure above 170 mmHg, known CV disease and albuminuria. Abbreviations: MR-proANP, Mid-regional pro-atrial natriuretic peptide; HFpEF, heart failure with preserved ejection fraction; HFrEF, heart failure with reduced ejection fraction.

**Additional file 1: Table S2:** Associations between MR-proANP and echocardiographic measures.

|  | Univariable | | Multivariable | |
| --- | --- | --- | --- | --- |
|  | Coefficient  (standard error) | p-value | Coefficient  (standard error) | p-value |
| Left ventricular mass index, g/m^2^ | 7.1 (0.8) | <0.0001 | 7.5 (2.3) | 0.002 |
| Interventricular septum thickness, mm | 0.3 (0.1) | <0.0001 | 0.1 (0.2) | 0.7 |
| Left ventricular internal diameter in end-diastole, mm | 0.7 (0.2) | 0.002 | 1.8 (0.6) | 0.006 |
| Inferolateral wall thickness, mm | 0.3 (0.1) | <0.0001 | 0.3 (0.2) | 0.1 |
| Left atrial volume index, ml/m^2^ | 3.7 (0.3) | <0.0001 | 5.0 (0.8) | <0.0001 |
| Early diastolic mitral inflow velocity (E), m/sec | 0.04 (0.01) | <0.0001 | 0.1 (0.02) | 0.0006 |
| Atrial mitral inflow velocity (A), m/sec | 0.04 (0.01) | <0.0001 | 0.01 (0.02) | 0.6 |
| E/A ratio | 0.01 (0.01) | 0.3 | 0.07 (0.02) | 0.003 |
| Mitral valve deceleration time, msec | 6.4 (2.7) | 0.02 | -8.1 (7.7) | 0.3 |
| Early diastolic lateral annular velocity (e’_lateral_), cm/sec | -0.7 (0.1) | <0.0001 | -0.05 (0.2) | 0.8 |
| Early diastolic septal annular velocity (e’ _septal_), cm/sec | -0.7 (0.1) | <0.0001 | -0.4 (0.2) | 0.03 |
| E/e’ _mean_ | 1.8 (0.2) | <0.0001 | 1.8 (0.4) | <0.0001 |
| Left ventricular ejection fraction, biplane, % | -1.1 (0.3) | 0.0007 | -1.8 (0.9) | 0.04 |
| Left ventricular global longitudinal strain, % | 0.3 (0.1) | 0.005 | 0.4 (0.3) | 0.2 |

Coefficients are per doubling of mid-regional pro-atrial natriuretic peptide (MR-proANP). Adjustments in the multivariable analysis include age, sex, duration of type 2 diabetes, known cardiovascular disease, systolic blood pressure above 170 mmHg, body mass index, and albuminuria.
